# Decoupling Principle Analysis and Development of a Parallel Three-Dimensional Force Sensor

**DOI:** 10.3390/s16091506

**Published:** 2016-09-15

**Authors:** Yanzhi Zhao, Leihao Jiao, Dacheng Weng, Dan Zhang, Rencheng Zheng

**Affiliations:** 1 Key Laboratory of Parallel Robot and Mechatronic System of Hebei Province, Yanshan University, Qinhuangdao 066004, China; 18713509981@163.com (L.J.); wengdacheng409@163.com (D.W.); 2 Key Laboratory of Advanced Forging & Stamping Technology and Science of Ministry of Education of China, Yanshan University, Qinhuangdao 066004, China; 3 Department of Mechanical Engineering, Lassonde School of Engineering, York University, 4700 Keele Street, Toronto, ON M3J 1P3, Canada; dan.zhang@lassonde.yorku.ca; 4 Institute of Industrial Science, The University of Tokyo, Tokyo 153-8505, Japan; topzrc@iis.u-tokyo.ac.jp

**Keywords:** three-dimensional force sensor, mechanical decoupling, parallel structure, calibration experiment

## Abstract

In the development of the multi-dimensional force sensor, dimension coupling is the ubiquitous factor restricting the improvement of the measurement accuracy. To effectively reduce the influence of dimension coupling on the parallel multi-dimensional force sensor, a novel parallel three-dimensional force sensor is proposed using a mechanical decoupling principle, and the influence of the friction on dimension coupling is effectively reduced by making the friction rolling instead of sliding friction. In this paper, the mathematical model is established by combining with the structure model of the parallel three-dimensional force sensor, and the modeling and analysis of mechanical decoupling are carried out. The coupling degree (*ε*) of the designed sensor is defined and calculated, and the calculation results show that the mechanical decoupling parallel structure of the sensor possesses good decoupling performance. A prototype of the parallel three-dimensional force sensor was developed, and FEM analysis was carried out. The load calibration and data acquisition experiment system are built, and then calibration experiments were done. According to the calibration experiments, the measurement accuracy is less than 2.86% and the coupling accuracy is less than 3.02%. The experimental results show that the sensor system possesses high measuring accuracy, which provides a basis for the applied research of the parallel multi-dimensional force sensor.

## 1. Introduction

With the ability of measuring force components and torque components, the multi-dimensional force sensor is one of the most important and challenging kinds of sensors, widely applied in many research areas such as wind tunnel balances, thrust stand testing of rocket engines, and in robotics, automobile industry, aeronautics, etc. [1]. Some scholars have focused on the development of the multi-dimensional force sensor. Nguyen et al. [2] developed a Stewart platform-based sensor with LVDT’s mounted along the legs for force/torque measurement in the presence of passive compliance. Experiments conducted to evaluate the sensing capability of the force sensor were carried out and the results showed that it was capable of measuring torque with good performence. Stoughton [3] invented a modified Stewart platform manipulator, and the measurement accuracy and dexterity were all improved. Kerr [4] suggested that the Stewart platform with instrumented elastic legs can be used as a six-axis force sensor, and designed a Stewart-platform transducer. A simple illustrative example was given. Yao et al. [5] proposed a kind of six-dimensional force sensor based on the Stewart platform, and the spatially isotropic configuration is carried out. Ranganath et al. [6] designed a force/torque sensor based on a Stewart platform in a near-singular configuration from the angle of the sensor sensitivity. Beccai et al. [7] developed a hybrid silicon three-dimensional force sensor for biomechanical applications. Liu et al. [8] put forward a parallel six-dimensional heavy force/torque sensor based on the Stewart structure, and the sensor has a large measurement range, and good linearity. A kind of three-dimensional force sensor obtained by a novel alkaline etching technique was proposed by Va’zsonyi et al. [9], and the sensor showed good agreement with the theoretical predictions. GabSoon et al. [10] put forward a two-dimensional force sensor for measuring arm force of an upper-limb rehabilitation robot, and the sensor possessed a small interference error and repeatability error. Fontana et al. [11] developed a three-dimensional force sensor for dual finger haptic interfaces. Optimal design and experiment research of a fully pre-stressed six-dimensional force/torque sensor are done by Wang et al. [12], and the optimal design method and the superiority of the sensor structure were proved. The measurement accuracy is improved. Tsai et al. [13] designed a isotropic 6-DOF parallel manipulator using isotropy generators, and the sensor has a good measurement accuracy. Gao and Jin et al. [14,15] introduced the sensor to the miniaturization field by making elastic ball joints instead of spherical joints, and integral machining was realized. Yao et al. [16] proposed a fault-tolerant fully pre-stressed parallel six-dimensional force sensor, and a measurement theory and experimental study were carried out. The studies provided a basis for applied research on the fault-tolerant sensor. Zhao et al. [17] put forward a over-constrained 12-SS six-dimensional force sensor structure, and the simulation analysis is carried out. The above studies have mainly focused on the aspects concerning the structure design of the multi-dimensional force sensor, and some valuable results were obtained. However, dimension coupling is a ubiquitous factor restricting the improvement of the measurement accuracy.

How to effectively reduce the influence of dimension coupling on a parallel multi-dimensional force sensor is always been the key point, and some studies were carried out. Kang and Lee et al. [18] proposed a numerical shape optimization design procedure with effective representation and minimization of the cross coupling term. The multiple constraints on good isotropic measurement and safety were considered, and the cross coupling error was effectively reduced. Song et al. [19] put forward a four degree-of-freedom wrist force/torque sensor with an elastic body structure. The proposed sensor had low cross sensitivity. Elom et al. [20] introduced the decoupling parallel model to the design of the soft sensors, and developed a kind of soft sensor. The decoupling parallel model however needs further optimization. Chao et al. [21] carried out a study on the shape optimal design and force sensitivity evaluation of six-axis force sensors. The measurement accuracy was effectively improved. Liu et al. [22] developed a eight beams spoke six-dimensional force sensor, and both decoupling properties and a decoupling algorithm were developed, but the dimensional decoupling effect of the multi-dimensional force sensor was yet to be improved. Hou et al. [23] carried out a study on performance analysis and comprehensive index optimization of a new Stewart six-component force sensor configuration. The research results were fine, and useful for further study. Yu et al. [24] carried out a study on nonlinear static decoupling of a multi-dimensional force sensor based on BP and RBF neural networks and put forward a new method which can reduce the coupling effect. Dynamic experiments, modeling and compensation of a bar-shaped strain gauge balance for a wind tunnel were carried out by Xu et al. [25]. Liu et al. [26] researched static decoupling for multi-dimensional wheel force sensor, and the decoupling effect was not ideal. Xu et al. [27] proposed a solution to the analysis with cross-coupling matrix of six-dimensional wrist force sensor for Robot. Zhao et al. [28,29] put forward a new six-dimensional force sensor nonlinear static decoupling method combined with the advantages of hybrid hierarchy genetic algorithm, and the wavelet neural network was proposed to improve the measurement accuracy of large range six-dimensional force sensor. The analysis result showed that the method worked. These studies about sensor decoupling mainly focused on the structure optimization of the multi-dimensional force sensor and decoupling algorithm, and the certain decoupling effects were achieved, but dimension coupling still exists and restricts the improvement of the measurement accuracy of the multi-dimensional force sensor.

In this paper, innovation of the original design of the sensor structure is put forward. A kind of parallel three-dimensional force sensor is proposed using the mechanical decoupling principle. By making the friction rolling instead of sliding friction, the dimension coupling is effectively reduced and the measurement accuracy can be improved. In the same time, due to the parallel structure, the proposed kind of parallel three-dimensional force sensor can be used in multi-dimensional force measurements. A prototype of the proposed sensor is designed and fabricated. Then the load calibration and data acquisition experiment system are built, and the FEM analysis and the calibration experiments were done.

The organization of this paper is as follows: following the introduction, the structure of the sensor, the mathematical model and decoupling principle of the sensor are shown in Section 2. Section 3 analyses the decoupling characteristics of the sensor. Section 4 introduces the sensor prototype, the FEM analysis and the experimental study of static calibration of the prototype. The paper is concluded in Section 5, summarizing the present work.

## 2. Mathematical Model and Decoupling Principle

### 2.1. Structure

**Figure 1:**
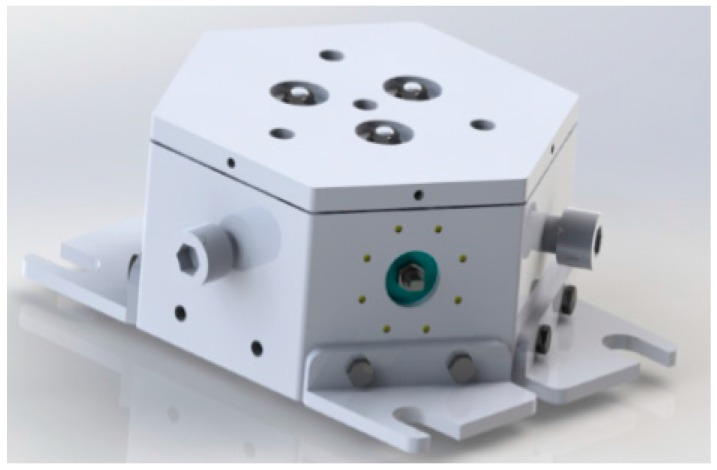
Design model of the parallel three-dimensional force sensor.

In order to effectively reduce the influence of dimension coupling on a parallel multi-dimensional force sensor, and improve the measurement accuracy of the sensor, a kind of parallel three-dimensional force sensor is proposed. The proposed sensor can measure three axial forces as x-axis, y-axis and z-axis, and the designed model is shown in Figure 1.

**Figure 2:**
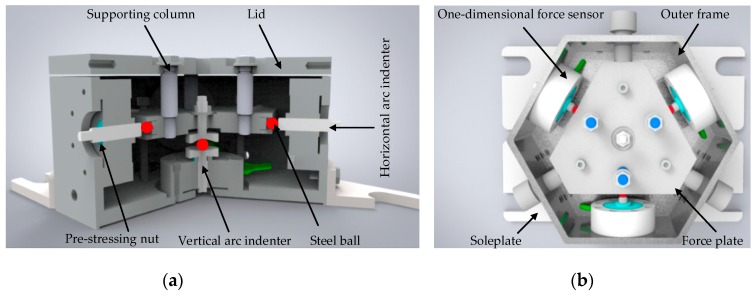
Internal structure of the parallel three-dimensional force sensor. (**a**) The internal structure of the sensor; (**b**) The top view of the internal structure.

The sensor mainly consists of a lid, a frame, a force plate and four force branches. The four force branches include three horizontal branches and a vertical branch. Each branch consists of a single-dimensional force sensor, arc indenters and a steel ball. The three horizontal branches uniformly distribute around the force plate and the vertical force branch is located just below the force plate. Each branch is composed of a single-dimensional force sensor, arc indenters and a steel ball. The force branches and the force plate are connected by the steel ball. The internal structure of the sensor is shown in Figure 2.

### 2.2. Measurement Model

**Figure 3:**
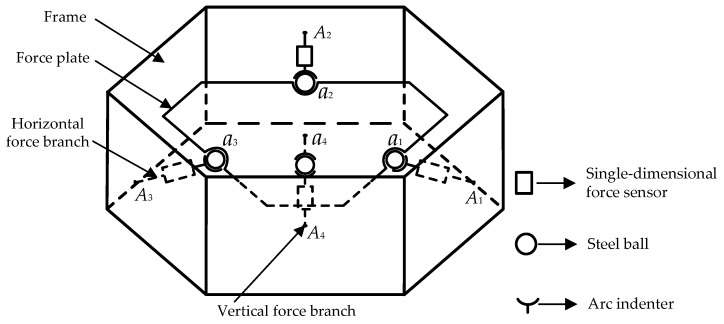
Schematic diagram of the parallel three-dimensional force sensor.

Figure 3 illustrates the diagram of the mechanical decoupling parallel three-dimensional force sensor, there exists mutual weak coupling between the force branches of the parallel three-dimensional force sensor. 

The symbols in Figure 3 are defined as follows: *a*_1_, *a*_2_, *a*_3_ and *a*_4_ are the contact points between the steel ball and force plate, *A*_1_, *A*_2_, *A*_3_ and *A*_4_ are the contact points between the force branch and the frame.

**Figure 4:**
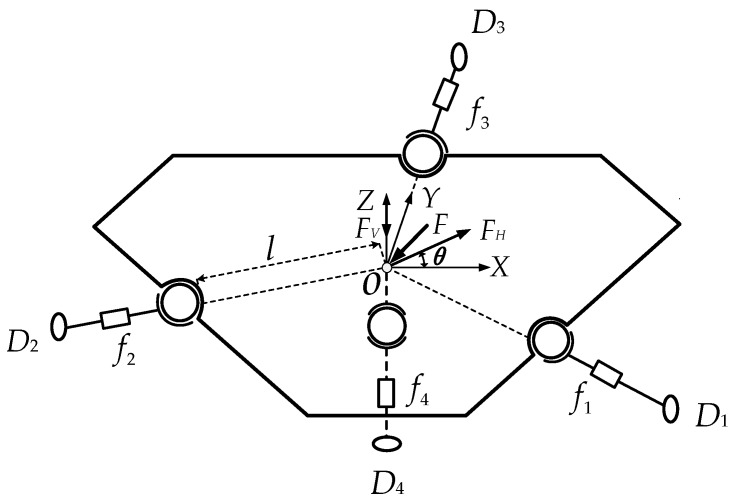
Force decomposition diagram of the sensor.

When there is an external force (*F*) acting on the force plate, the external force can be decomposed into a horizontal force (*F_H_*) and a vertical force (*F_V_*) as shown in Figure 4. The Cartesian coordinate system *O-XYZ* is set up with its origin located at the center of the force plate. *F* is the external force acting on the force plate; *f*_1_, *f*_2_, *f*_3_ and *f*_4_ are the measuring forces of the single-dimensional force sensor; *F_H_* is the horizontal component of the external force; *F_V_* is the vertical component of the external force, *D_i_* (*i* = 1, 2, 3, 4) denotes the *i*th branch of the sensor.

The equilibrium equation can be written according to the force diagram of the force plate with neglect of the influence of the friction:

(1) $$\left\{ \begin{array}{l} F_{X}-f_{1}\cos30^{{}^{\circ}}+f_{2}\cos30^{{}^{\circ}}=0\\ F_{Y}+f_{1}\cos60^{{}^{\circ}}+f_{2}\cos60^{{}^{\circ}}-f_{3}=0\\ F_{Z}-f_{4}=0 \end{array}\right.$$

where *F_X_*, *F_Y_* and *F_Z_* are the forces along coordinate axes, and *F_Z_* is equal to *F_V_*.

According to the structure of the sensor as shown in Figure 4, we can have:

(2) $$\left\{ \begin{array}{l} f_{1}\cos30^{{}^{\circ}}-F_{H}\cos\theta -f_{2}\cos30^{{}^{\circ}}=0\\ f_{1}\sin30^{{}^{\circ}}+F_{H}\sin\theta +f_{2}\sin30^{{}^{\circ}}-f_{3}=0 \end{array}\right.$$

where *θ* is the angle between *F_H_* and the positive direction of *X*-axis, as shown in Figure 4.

The relationship between *f*_1_, *f*_2_ and *f*_3_ can be obtained as:

(3) $$f_{3}=f_{1}(\sin30^{{}^{\circ}}+\cos30^{{}^{\circ}}\tan\theta )+f_{2}(\sin30^{{}^{\circ}}-\cos30^{{}^{\circ}}\tan\theta )$$

Substituting Equation (3) into Equation (1), the equilibrium equation can be rewritten as:

(4) $$\left\{ \begin{array}{l} F_{X}-f_{1}\cos30^{{}^{\circ}}+f_{2}\cos30^{{}^{\circ}}=0\\ F_{Y}-f_{1}\cos30^{{}^{\circ}}\tan\theta +f_{2}\cos30^{{}^{\circ}}\tan\theta =0\\ F_{Z}-f_{4}=0 \end{array}\right.$$

Equation (4) can be rewritten in the form of matrix equation as:

(5) $$F=\left[\begin{array}{l} F_{X}\\ F_{Y}\\ F_{Z} \end{array}\right]=Gf=\left[\begin{array}{ccc} \cos30^{{}^{\circ}} & -\cos30^{{}^{\circ}} & 0\\ \cos30^{{}^{\circ}}\tan\theta & -\cos30^{{}^{\circ}}\tan\theta & 0\\ 0 & 0 & 1 \end{array}\right]\left[\begin{array}{l} f_{1}\\ f_{2}\\ f_{3} \end{array}\right]$$

### 2.3. Force Model of Branch

The parallel three-dimensional force sensor was developed with the mechanical decoupling structure. Branch mechanical decoupling is realized depending on the rolling steel ball when there is an external force acting on the sensor, so the model and analysis of the force branch must be established based on the rolling friction law [30].

**Figure 5:**
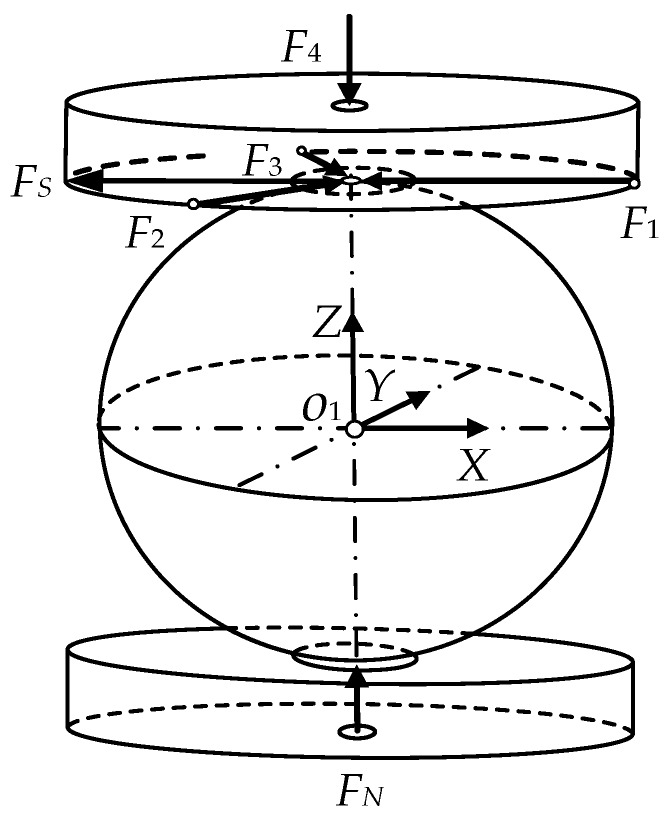
Force model of vertical branch.

The acting force diagram of the vertical force branch is shown in Figure 5. The branch steel balls of the parallel three-dimensional force sensor keep a balance under the arc indenter force, and the model can be simplified through projecting to x-z plane.

*F*_1_, *F*_2_ and *F*_3_ are the acting forces on the upper arc indenter along the direction of the three horizontal branches; *F*_4_ is the force acting on the upper arc indenter; *F*_N_ is the normal binding force; *F*_S_ is the summation of forces along the horizontal directions; *O*_1_*-XYZ* are the center coordinates.

**Figure 6:**
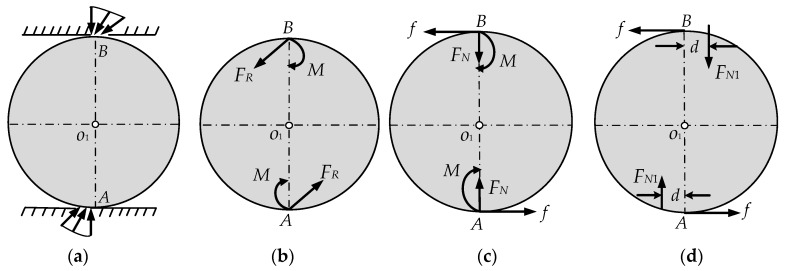
Force decomposition diagram of the steel ball. (**a**) The real force diagram of the ball; (**b**) The simplified force diagram of the sensor; (**c**) The decomposition diagram of (b); (**d**) The equivalent offset diagram of the steel ball.

Because the steel ball and the arc indenter are not actually a rigid body, they will deform under the acting force. The real force diagram of the ball is shown in Figure 6a. Through the reduction to point A, the distributed contact forces on the contact surface can be decomposed into a bearing reaction force and a force couple which is called couple of rolling resistance [30], and the simplified result is shown in Figure 6b. The bearing reaction force can be decomposed into a friction force and a normal binding force as shown in Figure 6c.

The couple of rolling resistance increases with the increase of the acting force, and the ball is going to be in a critical rolling state of equilibrium when the friction force increases with the external force to a certain value. At this moment, the couple of rolling resistance reaches the maximum value which is called maximum rolling friction couple [30]. The steel ball will roll if friction force increases again. Experiments show that the maximum rolling friction couple has nothing to do with the sphere radius, but it is proportional to the size of the normal binding force as shown in Equation (6):

(6) $$M=\delta F_{N}$$

where *M* is the couple of rolling resistance, *δ* is a proportionality constant which is called coefficient of rolling friction.

When the steel ball is in a critical state of equilibrium, the normal binding force and the maximum rolling friction couple can be synthesized into a off-center force based on the theorem of translation. The off-center force is equal to the normal binding force, and the offset distance is *d*, as shown in Figure 6d:

(7) $$d=M/F_{N1}=M/F_{N}$$

where *F_N_*_1_ is the off-center force, and *d* is the offset distance.

Combining Equation (6) with Equation (7), the relationship between the proportionality constant and the offset distance can be obtained as shown in Equation (8):

(8) δ=d

### 2.4. Force Analysis of the Rolling Steel Ball

According to Section 2.1, the steel ball and the arc indenter will deform under the acting force, but under the action of the friction on the contact surface, sliding cannot happen. To replace the deformation, the upper and lower arc indenter will produce a small tangential displacement, so the steel ball will produce a slight rotation relative to the horizontal plane before the critical rolling state.

**Figure 7:**
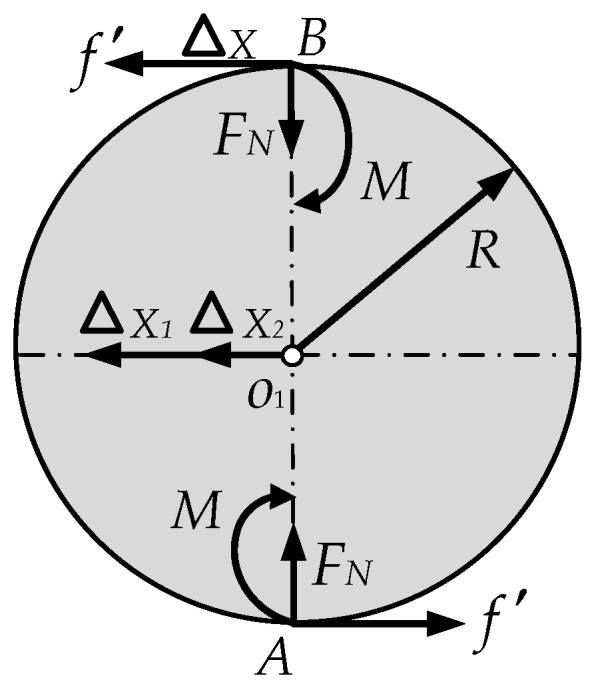
Force analysis diagram of the critical rolling steel ball.

In non-critical rolling state, the micro displacement of the steel ball center relative to the ground is $\Delta x_{1}$, and the micro displacement relative to the upper arc indenter is $\Delta x_{2}$, the micro displacement of the upper arc indenter relative to the ground is Δx, as shown in Figure 7, the relationship between $\Delta x_{1}$, $\Delta x_{2}$, and Δx can be expressed as shown in Equation (9):

(9) $$\Delta_{X}=\Delta_{X_{1}}+\Delta_{X_{2}}=2\Delta_{X_{1}}$$

Assuming that the relationship of the deformation in non-critical rolling state is linear, we can have:

(10) $$\Delta_{omax}/\Delta_{o}=\delta /d$$

where $\Delta_{omax}$ is the maximum displacement of the ball center in critical rolling state, $\Delta_{o}$ is the displacement of the ball center in non-critical rolling state.

For the equilibrium of the steel ball, the following equation can be obtained as:

(11) $$M=\;f^{\prime }R$$

Substituting Equations (7) and (10) into Equation (11), the friction force of the steel ball in non critical rolling state can be obtained as:

(12) $$f^{\prime }=(\delta F_{N}/(R\Delta_{XMAX}))\Delta_{X}$$

where *f*′ is the friction force of the steel ball, $\Delta_{XMAX}$ is the maximum displacement of the upper arc indenter relative to the ground in critical rolling state, *R* is the steel ball radius, and $\Delta_{X}$ is the displacement of the upper arc indenter relative to the ground in non-critical rolling state.

### 2.5. Rolling Friction Experiment

**Figure 8:**
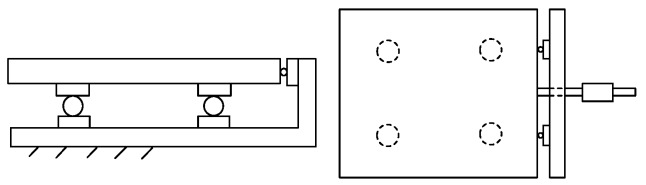
The rolling friction experiment system model.

In order to prove that the rolling motion has a fine decoupling effect, a experiment has been carried out. The experiment system model is shown in Figure 8.

**Figure 9:**
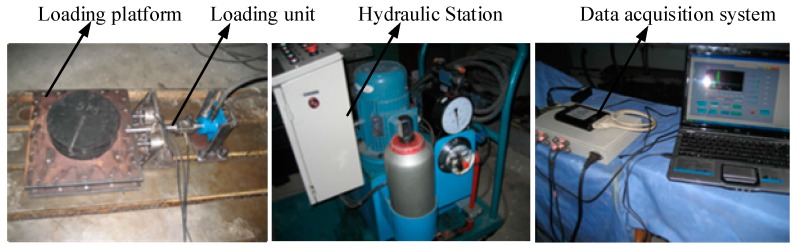
The rolling friction experiment system.

The experiment system is established as shown in Figure 9, which consists of a loading platform, a loading unit, a hydraulic station and a data acquisition system. The loading unit is composed of a loading rack, a loading cylinder, a tension sensor and a pull rod. Two single-dimensional force sensors are installed on the loading rack, which are used for the force measurement.

In the experiment, different weights were put on the platform, and different forces loaded by the loading unit. The weights include 0 kg, 25 kg, 106.8 kg and 176.1 kg. The experimental data are shown in Table 1, Table 2, Table 3 and Table 4.

**Figure 10:**
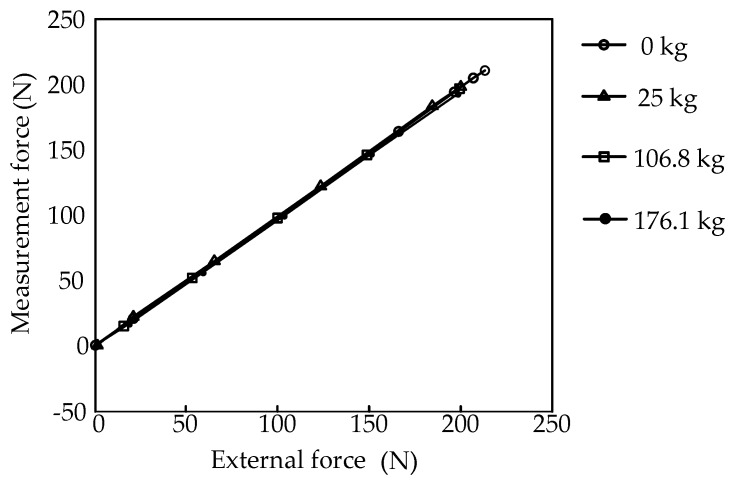
Force diagram with consideration of friction.

According to the above data, the relationship between the loading force and the measurement force can be obtained as shown in Figure 10.

The experimental results shows that the vertical weights loaded on the platform have little influence on the horizontal measurement accuracy, and the rolling motion has a fine decoupling measurement effect. Therefore, the feasibility of the mechanical decoupling principle of the parallel three-dimensional force sensor proposed in this paper is proved.

### 2.6. Mechanical Decoupling Principle

**Figure 11:**
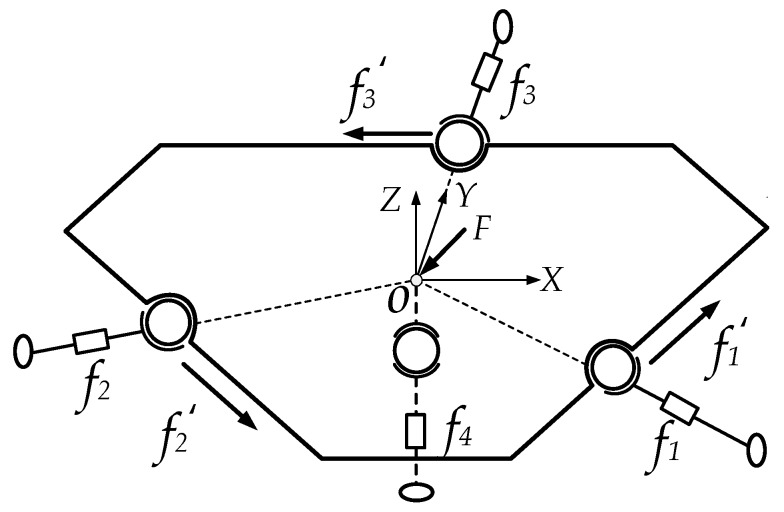
Force diagram with consideration of friction.

The influence of coupling between branches of the parallel three-dimensional force sensor can be reduced by making the rolling friction instead of sliding friction. The force diagram is shown in Figure 11, and the equilibrium equation of the force plate can be established as shown in Equation (13).

(13) $$\left\{ \begin{array}{l} F_{X}-C_{1}f_{1}^{\prime }\cos60^{{}^{\circ}}-C_{2}f_{2}^{\prime }\cos60^{{}^{\circ}}-f_{3}^{\prime }-f_{4}^{\prime }-f_{1}\cos30^{{}^{\circ}}+f_{2}\cos30^{{}^{\circ}}=0\\ F_{Y}-C_{3}f_{1}^{\prime }\cos30^{{}^{\circ}}-C_{4}f_{2}^{\prime }\cos30^{{}^{\circ}}-f_{4}^{\prime }-C_{5}f_{1}\cos60^{{}^{\circ}}-C_{5}f_{2}\cos60^{{}^{\circ}}+C_{5}f_{3}=0\\ F_{Z}-f_{1}^{\prime }-f_{2}^{\prime }-f_{3}^{\prime }-f_{4}^{\prime }=0 \end{array}\right.$$

where *C_i_* denotes the direction coefficient of the friction, whose value depends on the direction of the force components in horizontal plane, and its value may be ±1 or 0; $f_{1}^{\prime }$, $f_{2}^{\prime }$, $f_{3}^{\prime }$ and $f_{4}^{\prime }$ are the friction forces of the steel balls.

The friction force along different branches is influenced by the manufacturing and machining accuracy, so assuming that the steel balls are all in the critical rolling state, and the friction force reaches the maximum value. The following relationship can be derived according to the Equation (12):

(14) $$f_{1}^{\prime }=\delta f_{1}/R;f_{2}^{\prime }=\delta f_{2}/R;f_{3}^{\prime }=\delta f_{3}/R;f_{4}^{\prime }=\delta f_{4}/R$$

Substituting Equation (14) into Equation (13), the equilibrium equation can be obtained as:

(15) $$\left[\begin{array}{l} F_{X}\\ F_{Y}\\ F_{Z} \end{array}\right]=\left[\begin{array}{ccc} \frac{C_{1}\delta \cos60^{{}^{\circ}}}{R}+\cos30^{{}^{\circ}} & \frac{C_{2}\delta \cos60^{{}^{\circ}}}{R}-\cos30^{{}^{\circ}} & \begin{array}{cc} \frac{\delta }{R} & \frac{\delta }{R} \end{array}\\ \frac{C_{3}\delta \cos30^{{}^{\circ}}}{R}+C_{5}\cos60^{{}^{\circ}} & \frac{C_{4}\delta \cos30^{{}^{\circ}}}{R}+C_{5}\cos60^{{}^{\circ}} & \begin{array}{cc} -C_{5} & \frac{\delta }{R} \end{array}\\ \frac{\delta }{R} & \frac{\delta }{R} & \begin{array}{cc} \frac{\delta }{R} & \frac{\delta }{R} \end{array} \end{array}\right]\left[\begin{array}{c} f_{1}\\ \begin{array}{c} f_{2}\\ f_{3}\\ f_{4} \end{array} \end{array}\right]$$

The relationship between *f*_1_, *f*_2_ and *f*_3_ can be derived and simplified as shown in Equation (16) according to the force diagrams in Figure 11.

(16) $$f_{3}=C_{1}f_{1}+C_{2}f_{2}$$

Substituting Equation (16) into Equation (13), the equilibrium equation can be simplified as:

(17) $$\left\{ \begin{array}{l} F_{X}-[\frac{3}{2}\frac{C_{1}\delta }{R}+\cos30^{{}^{\circ}}]f_{1}-[\frac{3}{2}\frac{C_{1}\delta }{R}-\cos30^{{}^{\circ}}]f_{2}-\frac{\delta }{R}f_{4}=0\\ F_{Y}-[\frac{C_{3}\delta \cos30^{{}^{\circ}}}{R}+(\frac{1}{2}-C_{1})C_{5}]f_{1}-[\frac{C_{4}\delta \cos30^{{}^{\circ}}}{R}+((\frac{1}{2}-C_{2})C_{5})]f_{2}-\frac{\delta }{R}f_{4}=0\\ F_{Z}-[\frac{\delta }{R}+C_{1}]f_{1}+[\frac{\delta }{R}+C_{2}]f_{2}-f_{4}=0 \end{array}\right.$$

The the equilibrium equation can be rewritten in another form of matrix equation as:

(18) $$F=\left[\begin{array}{l} F_{X}\\ F_{Y}\\ F_{Z} \end{array}\right]=Gf=\left[\begin{array}{ccc} \frac{3}{2}\cdot \frac{C_{1}\delta }{R}+\cos30^{{}^{\circ}} & \frac{3}{2}\cdot \frac{C_{1}\delta }{R}-\cos30^{{}^{\circ}} & \frac{\delta }{R}\\ \frac{C_{3}\delta \cos30^{{}^{\circ}}}{R}+(\frac{1}{2}-C_{1})C_{5} & \frac{C_{4}\delta \cos30^{{}^{\circ}}}{R}+(\frac{1}{2}-C_{2})C_{5} & \frac{\delta }{R}\\ \frac{\delta }{R}+C_{1} & -\left(\frac{\delta }{R}+C_{2}\right) & 1 \end{array}\right]\left[\begin{array}{l} f_{1}\\ f_{2}\\ f_{4} \end{array}\right]$$

## 3. Decoupling Characteristic Analysis

Based on the above force analysis of the parallel three-dimensional force sensor, we assume that the angle between the external force component in the horizontal plane and *X*-axis is sixty degrees, and the coefficient of rolling friction is 0.01, and the steel ball radius is 10 mm. The relationship between the measuring force and the external force in the ideal case can be expressed as shown in Equation (19):

(19) $$\left[\begin{array}{l} f_{1}\\ f_{2}\\ f_{4} \end{array}\right]=\left[\begin{array}{ccc} 1.7321 & -1.0000 & 0.0000\\ 0.5774 & -1.0000 & 0.0000\\ 0.0000 & 0.0000 & 1.0000 \end{array}\right]\left[\begin{array}{l} F_{X}\\ F_{Y}\\ F_{Z} \end{array}\right]$$

**Figure 12:**
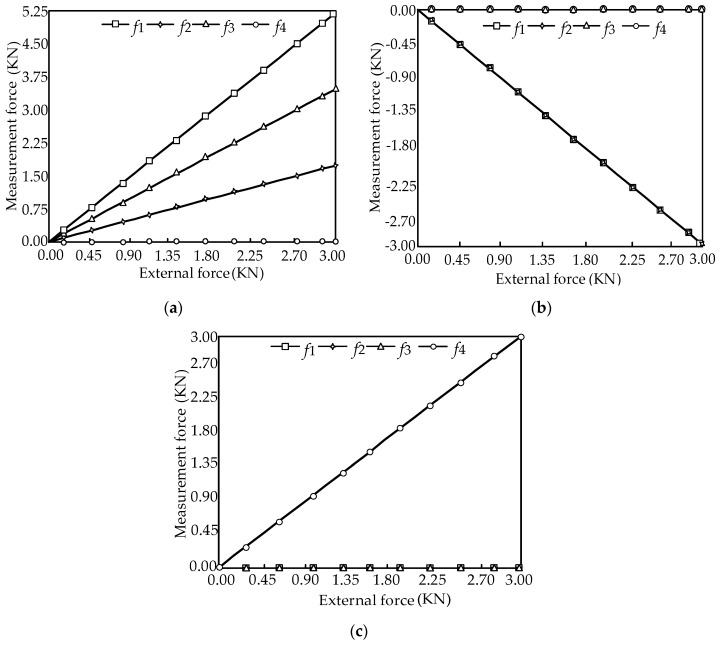
Force diagram of the sensor. (**a**) The relationships between the measurement forces *f_i_* (*i* = 1, 2, 3, 4) and the external force component *F_X_*; (**b**) The relationships between the measurement forces *f_i_* (*i* = 1, 2, 3, 4) and the external force component *F_Y_*; (**c**) The relationships between the measurement forces *f_i_* (*i* = 1, 2, 3, 4) and the external force component *F_Z_*.

When the external forces are limited to 0~3000 N, the force diagrams between the measurement forces and the external force in the ideal case are shown in Figure 12.

In Figure 12, it is shown that the change rule between the measurement force and external force is linear. Substituting the structure parameter (*θ* = 60°) into Equation (18), the equilibrium equation with consideration of friction can be obtained as:

(20) $$F=\left[\begin{array}{l} F_{X}\\ F_{Y}\\ F_{Z} \end{array}\right]=\left[\begin{array}{ccc} 0.8675 & -0.8675 & 0.0010\\ 0.5009 & -1.4991 & 0.0010\\ 0.0020 & 0.0000 & 1.0000 \end{array}\right]\left[\begin{array}{l} f_{1}\\ f_{2}\\ f_{4} \end{array}\right]=G^{\prime }f$$

where *G*′ is a introducing matrix which shows the numerical relation between the measurement force and the external force.

The Equation (20) can be rewritten in another form of equation as shown in Equation (21):

(21) $$\left[\begin{array}{l} f_{1}\\ f_{2}\\ f_{4} \end{array}\right]={G^{\prime }}^{-1}f=\left[\begin{array}{ccc} 1.7321 & -1.0018 & -0.0007\\ 0.5784 & -1.0018 & 0.0004\\ -0.0035 & 0.0020 & 1.0000 \end{array}\right]\left[\begin{array}{l} F_{X}\\ F_{Y}\\ F_{Z} \end{array}\right]$$

**Figure 13:**
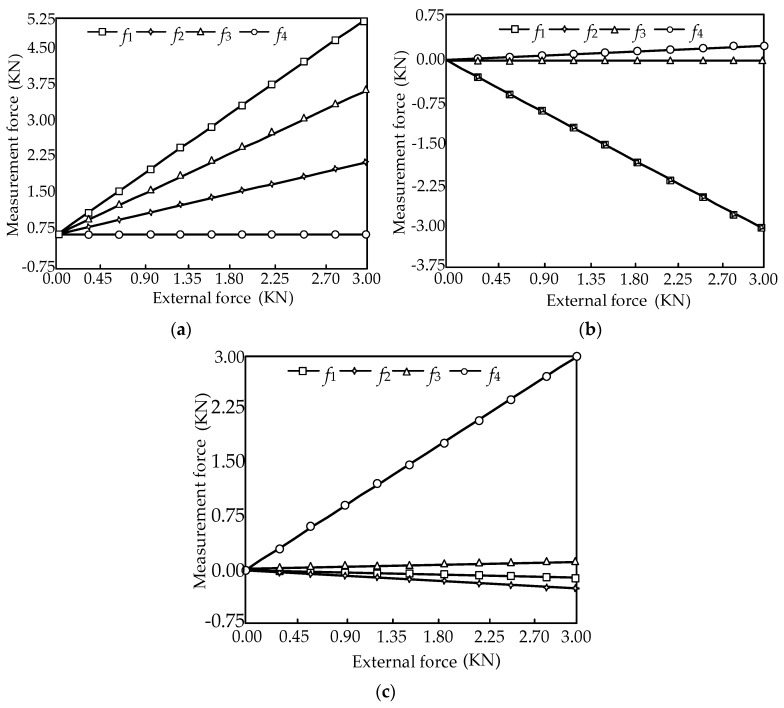
Force diagram of the sensor. (**a**) The relationships between the measurement forces *f_i_* (*i* = 1, 2, 3, 4) and the external force component *F_X_*; (**b**) The relationships between the measurement forces *f_i_* (*i* = 1, 2, 3, 4) and the external force component *F_Y_* (**c**) The relationships between the measurement forces *f_i_* (*i* = 1, 2, 3, 4) and the external force component *F_Z_*.

When the external forces are limited to 0~3000 N with consideration of friction, the force diagrams between the measurement forces and the external force are shown in Figure 13.

In Figure 13, it is shown that the change rule with consideration of friction is also linear, and the influence of friction is effectively reduced. The measurement force error in the ideal case and with consideration of friction can be expressed as:

(22) $$\Delta f=f_{L}-f_{M}$$

where Δf is the measurement force error, *f_L_* is the measurement force in the ideal case, *f_M_* is the measurement force with consideration of friction.

**Figure 14:**
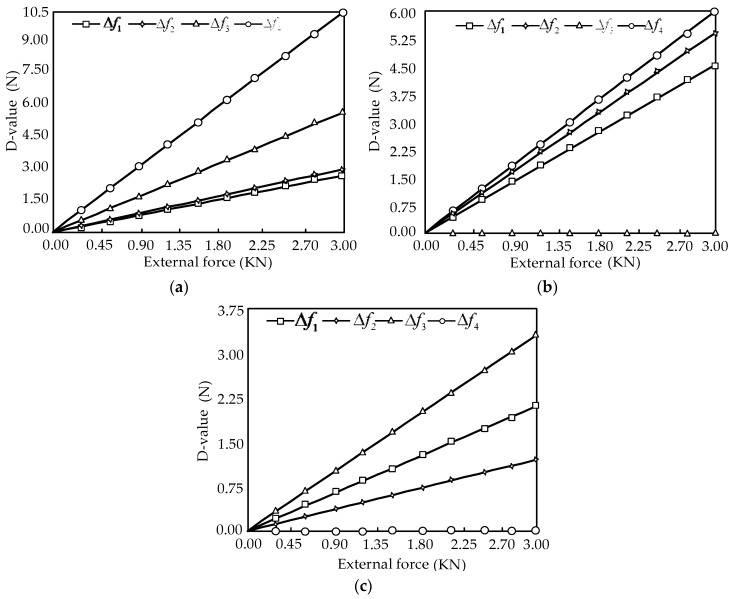
Relations of the force error and external force. (**a**) The relationships between the measurement force error $\Delta f_{i}$ (*i* = 1, 2, 3, 4) and the external force component *F_X_*; (**b**) The relationships between the measurement force error $\Delta f_{i}$ (*i* = 1, 2, 3, 4) and the external force component *F_Y_* (**c**) The relationships between the measurement force error $\Delta f_{i}$ (*i* = 1, 2, 3, 4) and the external force component *F_Z_*.

The force diagrams between the measurement force error (D-value) and the external force are shown in Figure 14.

In Figure 14, it is shown that the measurement force error is small, and the change rule is linear.

There is a certain coupling relationship between branches of the sensor, so the coupling degree (*ε*) can be defined as shown in Equation (23):

(23) $$\varepsilon =\frac{f_{L}-f_{M}}{f_{L}}$$

where *ε* is the defined coupling degree.

Assuming that the external force is 3000 N, the measurement forces and the coupling degrees are shown in Table 5.

According to the definition of coupling degree and the related calculation results as shown in Table 5, the integrated coupling degree of the sensor is less than 2.85%, so the designed parallel three-dimensional force sensor with the mechanical decoupling structure possesses good decoupling performance.

## 4. Calibration Experiment

### 4.1. Prototype

**Figure 15:**
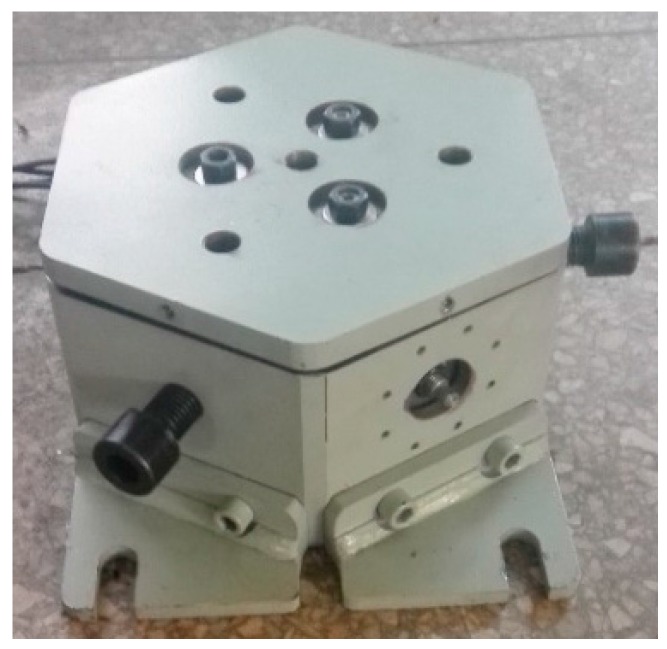
Prototype of the parallel three-dimensional force sensor.

A mechanical decoupling parallel three-dimensional force sensor prototype is developed based on the mathematic model and decoupling analysis, and it can measure three-dimensional space force. The size of the prototype is 365 × 257 × 142 mm, and the sensor prototype is shown in Figure 15.

Due to the application of the mechanical decoupling structure, the pre-stressed force is needed to ensure that all the branches are always in compression when subjected to the expected range of external force, and the pre-stressed force can be adjusted by pre-stressing the nut properly.

### 4.2. FEM Analysis

Before the calibration experiment, a FEM analysis is carried out to simulate the loading experiment. The simulation is carried out by 300 N interval. The mesh data of the sensor is shown in Table 6.

In the analysis, we assume that the force of the branch has a certain relationship with the axial deformation, and the measured axial deformation can be used instead of the force of the branch, so we set up coordinate system in the simulation software. 

**Figure 16:**
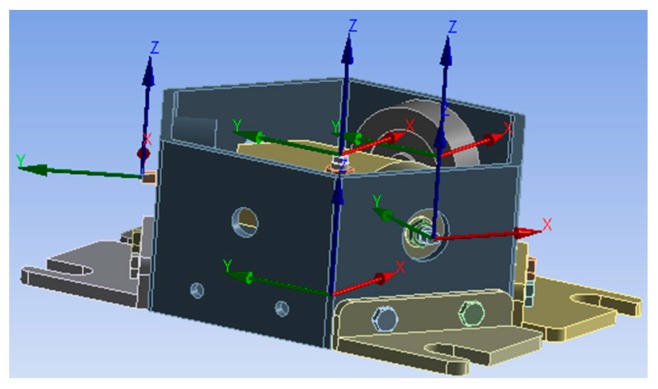
Coordinate system of the sensor.

**Figure 17:**
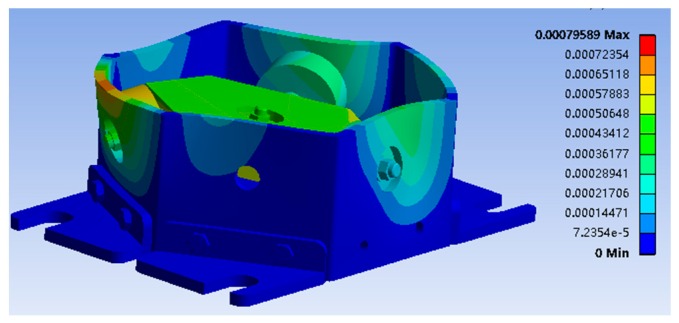
Deformation image of the sensor.

The probe point is the center of the sensor, and the coordinate origins of the single-dimensional force sensor of each branch are shown in Figure 16. When loading 2700 N along the x-axis, the deformation image is shown in Figure 17.

When loading force along x-axis ranges from 300 N to 3300 N, the axial deformations of the four branches are shown in Table 7. 

When loading along z-axis, the probe point is the center of the sensor, and the coordinate origins of the single-dimensional force sensor of each branch are maintained. The axial deformations of the four branches are shown in Table 8.

**Figure 18:**
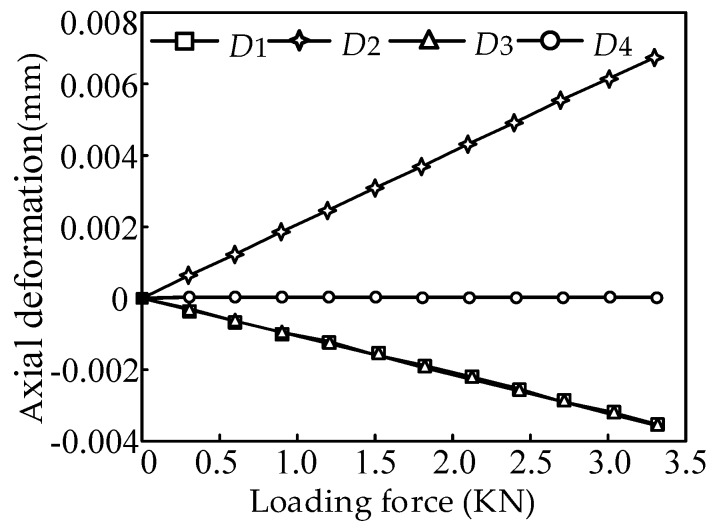
Axial deformations of the four branches.

When loading along x-axis and y-axis in the same time, the axial deformation of the four branches is shown in Figure 18.

In the analysis, the axial deformation of the four branches was measured. The force of the branch has a certain relationship with the axial deformation, and the measured axial deformation can be used instead of the force of the branch. Based on the least square method, the calibration matrix and the error matrix can be obtained by using the FEM analysis data:

(24) $$G=\left[\begin{array}{cccc} 1.0005 & 0.0018 & -0.0121 & -0.0109\\ -0.0012 & 1.0008 & 0.0035 & -0.0101\\ -0.0015 & -0.0032 & 1.0009 & -0.0156\\ -0.0035 & -0.0022 & -0.0002 & 1.0022 \end{array}\right]$$

(25) $$Err=\left[\begin{array}{cccc} 0.0014 & 0.0025 & 0.0105 & 0.0132\\ 0.0036 & 0.0098 & 0.0010 & 0.0196\\ 0.0042 & 0.0046 & 0.0096 & 0.0153\\ 0.0021 & 0.0056 & 0.0032 & 0.0006 \end{array}\right]$$

According to the physical meaning of the error matrix, the diagonal elements of the error matrix denote the measurement accuracies of the four branches, and the other elements denote the accuracy of coupling of the sensor, so the branch measurement accuracies of the parallel three-dimensional force sensor are 0.14%, 0.98%, 0.96% and 0.06%, and the accuracy of coupling is less than 1.96%. Because there is no machining error and assembly error, the accuracies are relatively high, and the results have a certain reference value.

### 4.3. Static Calibration

**Figure 19:**
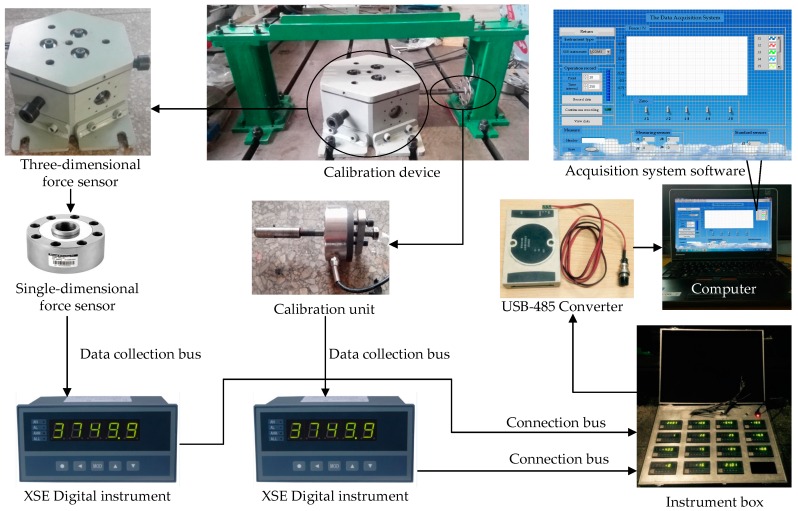
Flow diagram of the calibration system.

In order to calibrate the prototype of the parallel three-dimensional force sensor and verify each performance of the sensor, the static calibration experiment is established. The load calibration equipment and data acquisition experiment system of sensor are built, which are consists of high precision standard force sensor, the prototype of three-dimensional force sensor, intelligent instrument and computer. The flow diagram of the calibration system is shown in Figure 19.

**Figure 20:**
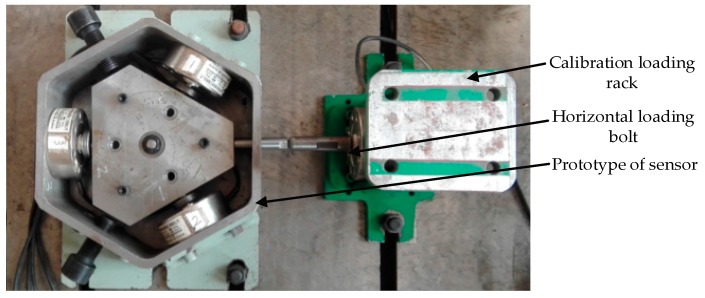
The calibration experiment of the horizontal loading.

**Figure 21:**
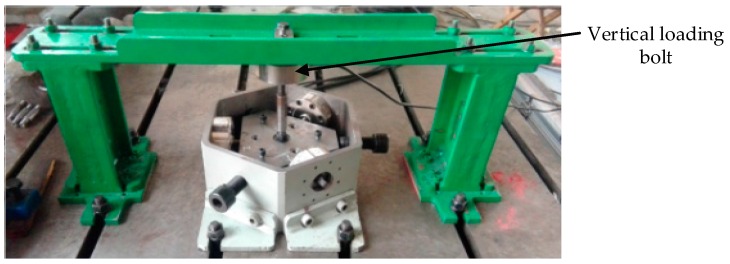
The calibration experiment of the vertical loading.

The static calibration experiment in this paper is carried out when the horizontal pre-stressed force is 500 N, and the measure range of the three-dimensional force sensor is 3000 N. Because the structure of the sensor is non-orthogonal, the calibration experiment must be carried out along the four branches. The load calibration experiment schematic diagrams of the vertical direction and the horizontal direction are shown in Figure 20 and Figure 21.

The calibration results which is deduced out with the least square method can be expressed as shown in Equation (26):

(26) $$F^{\prime }=G^{\prime \prime }f_{c}+E$$

where ***F***′ is the matrix of calibration loading with four rows and *n* columns, and *n* is the number of calibration force vectors, ***G***′′ is the calibration matrix with four rows and *n* columns, ***f****_c_* is the calibration measuring force with *n* rows and *n* columns, ***E*** is the error matrix with four rows and *n* columns.

The *i*th dimension of the calibration force vector can be obtained from the transpose of the *i*th rows of the calibration matrix, as shown in Equation (27):

(27) $$F_{i}^{\prime }={\left[\begin{array}{cccc} F_{1} & F_{2} & F_{3} & \begin{array}{cc} \cdot \cdot \cdot & F_{n} \end{array} \end{array}\right]}^{T}$$

The *i*th dimension of the calibration vector can be obtained from the transpose of the *i*th rows of the calibration matrix, as shown in Equation (28):

(28) $${G_{i}}^{\prime \prime }={\left[\begin{array}{cccc} {G_{1}}^{\prime \prime } & {G_{2}}^{\prime \prime } & {G_{3}}^{\prime \prime } & \begin{array}{cc} \cdot \cdot \cdot & {G_{n}}^{\prime \prime } \end{array} \end{array}\right]}^{T}$$

The *i*th dimension of the error vector can be obtained from the transpose of the *i*th rows of the error matrix, as shown in Equation (29):

(29) $$E_{i}={\left[\begin{array}{cccc} E_{1} & E_{2} & E_{3} & \begin{array}{cc} \cdot \cdot \cdot & E_{n} \end{array} \end{array}\right]}^{T}$$

Then, Equation (26) can be rewritten in the form of equation as shown in Equation (30):

(30) $$F_{i}^{\prime }=f_{c}^{T}G_{i}^{\prime \prime }+E_{i}$$

where $G_{i}^{\prime \prime }$ is the calibration vector, $F_{i}^{\prime }$ is the vector of calibration loading, ***E****_i_* is the error matrix vector.

Based on the the least square method, the following relationship shown in Equations (31) and (32) can be established:

(31) $$J_{i}=\sum_{j=1}^{n}{E_{ij}}^{2}=E_{i}^{T}E_{i}$$

(32) $${\frac{\partial J_{i}}{\partial G_{i}^{\prime \prime }}|}_{G_{i}^{\prime \prime }=\bar{G_{i}^{\prime \prime }}}={\frac{\partial {\left(F_{i}^{\prime }-f_{c}^{T}G_{i}^{\prime \prime }\right)}^{T}{\left(F_{i}^{\prime }-f_{c}^{T}G_{i}^{\prime \prime }\right)}^{T}}{\partial G_{i}^{\prime \prime }}|}_{G_{i}^{\prime \prime }=\bar{G_{i}^{\prime \prime }}}=0$$

where ***J****_i_* is a factor for evaluating the measuring error, $\bar{G_{i}^{\prime \prime }}$ is the augmented matrix. 

The inverse matrix of the matrix $f_{c}f_{c}^{T}$ exists when the matrix $f_{c}f_{c}^{T}$ is full rank, and the *i*th dimension of the calibration vector can be expressed as shown in Equation (33):

(33) $$\bar{G_{i}^{\prime \prime }}={\left(f_{c}f_{c}^{T}\right)}^{-1}f_{c}F_{i}^{\prime }$$

Because *i* = 4 in the calibration experiment, the calibration matrix can be deduced as shown in Equation (34).

(34) $$G^{\prime \prime }=\left[\begin{array}{cccc} \bar{{G_{1}}^{\prime \prime }} & \bar{{G_{2}}^{\prime \prime }} & \bar{{G_{3}}^{\prime \prime }} & \bar{{G_{4}}^{\prime \prime }} \end{array}\right]=F^{\prime }f_{c}^{T}{\left(f_{c}f_{c}^{T}\right)}^{-1}$$

The calibration matrix can be obtained as shown in Equation (35) when the horizontal pre-stressed force is 500 N.

(35) $$G^{\prime \prime }=\left[\begin{array}{cccc} 1.0025 & 0.0088 & -0.0148 & -0.0339\\ -0.0023 & 1.0067 & 0.0105 & -0.0402\\ -0.0025 & -0.0044 & 1.0018 & -0.0396\\ -0.0066 & -0.0060 & -0.0003 & 1.0312 \end{array}\right]$$

### 4.4. Calibrating Results

**Figure 22:**
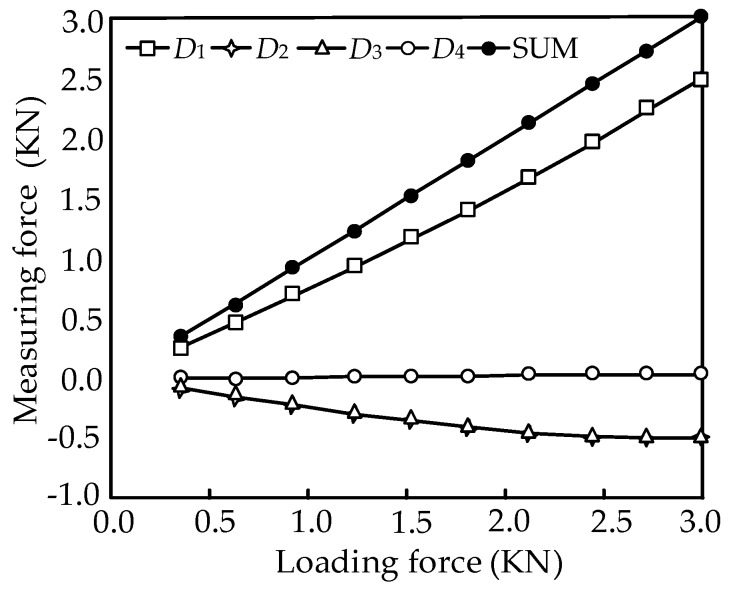
Calibration on the first branch.

**Figure 23:**
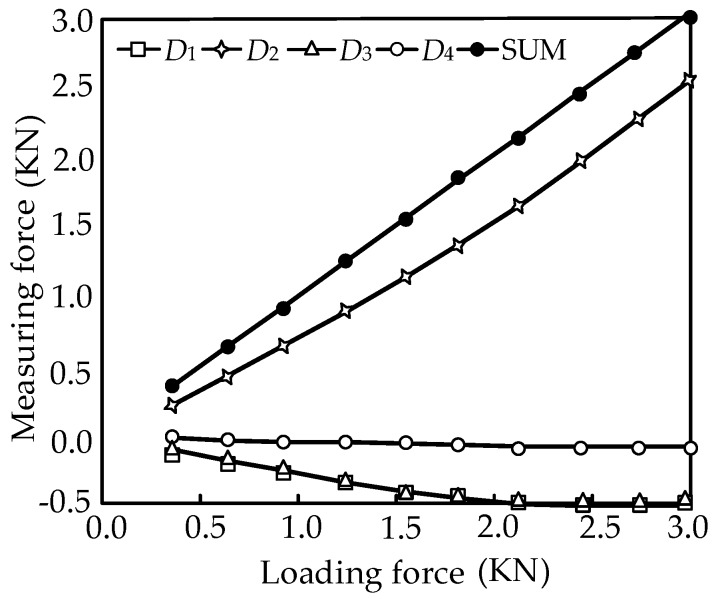
Calibration on the second branch.

**Figure 24:**
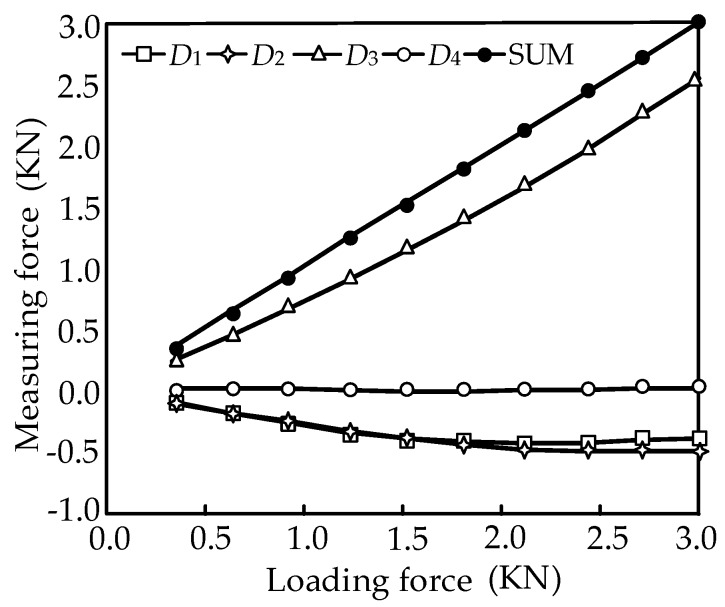
Calibration on the third branch.

**Figure 25:**
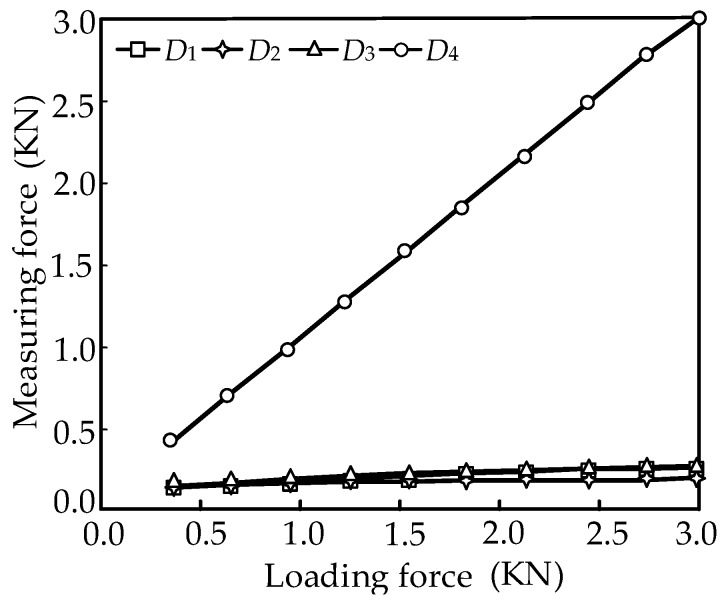
Calibration on the fourth branch.

The static calibration experiments are respectively carried out along the direction of the branches when the horizontal pre-stressed force is 500 N. *Di* (*i* = 1, 2, 3, 4) denotes the *i*th branch of the sensor as shown in Figure 3, and SUM is the total force of all the loading directions. The input and output force relations are respectively shown in Figure 22, Figure 23, Figure 24 and Figure 25. As shown in the four figures, the change rule of the total force is also linear, which confirms the coherence between the theoretical analyses and experiment results.

Loading in one direction, the sensor readings in the other directions will change, and the change is influenced by the pre-stressed force. Substituting the structure parameter and the the horizontal pre-stressed force into the error matrix, and the error matrix of the sensor can be obtained as:

(36) $$Err=\left[\begin{array}{cccc} 0.0052 & 0.0099 & 0.0225 & 0.0265\\ 0.0053 & 0.0189 & 0.0009 & 0.0301\\ 0.0059 & 0.0046 & 0.0286 & 0.0302\\ 0.0073 & 0.0070 & 0.0060 & 0.0026 \end{array}\right]$$

According to the error matrix, the branch measurement accuracies of the parallel three-dimensional force sensor are 0.52%, 1.89%, 2.86% and 0.26%, and the accuracy of coupling is less than 3.02%. The experimental results show that the parallel three-dimensional force sensor possesses good decoupling performance and high measuring accuracy.

**Figure 26:**
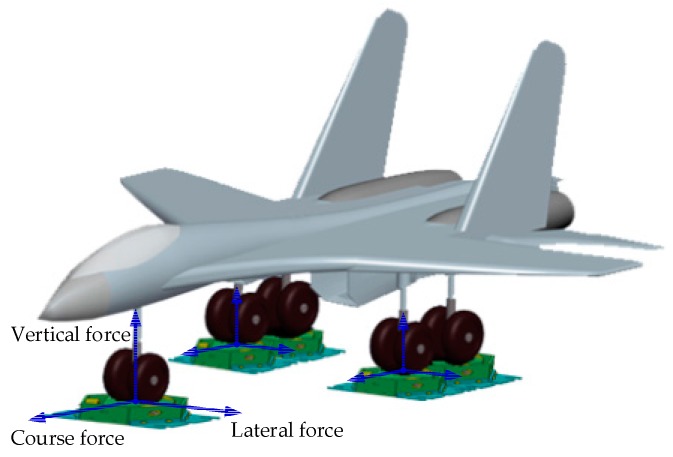
The landing gear load test experiment.

In the near future, further optimization of the sensor will be paid attention to, and the proposed sensor can be mostly used in the large range spacial three dimensional force measurement. For example, the proposed sensor can be applied in landing gear load test as shown in Figure 26, and the sensor can measure the vertical force, the lateral force and the course force of the airplane landing gear.

## 5. Conclusions

In this paper, to effectively reduce the influence of dimension coupling on a parallel multi-dimensional force sensor, a parallel three-dimensional force sensor is proposed and developed using the mechanical decoupling principle, and the mathematical model is established and analyzed. Aimed at the performance characteristics of the sensor, the definition of coupling degree is put forward and calculated, and the calculation results show that the designed sensor possesses good decoupling performance. FEM analysis was carried out, then the load calibration and data acquisition experiment system were built, and calibration experiments were done. The experiment results show that the measurement accuracy is less than 2.86%, and the accuracy of coupling is less than 3.02%. The experimental results agree with the theoretical calculation values, and the whole experiment achieves the desired effect and verifies each performance feature of the sensor.

## Figures and Tables

**Table 1:** Experiment 1 (the weight is 0 kg).

**Loading Force/N**	0	21.238	166.261	196.797	207.097	213.386
**Output Force 1/N**	0.123	9.844	81.831	96.967	102.166	105.273
**Output Force 2/N**	0	11.168	82.192	97.25	102.394	105.469

**Table 2:** Experiment 2 (the weight is 25 kg).

**Loading Force/N**	1.458	21.147	65.173	123.419	184.855	200.442
**Output Force 1/N**	−0.061	10.029	31.318	59.805	89.892	97.521
**Output Force 2/N**	0	11.921	33.692	62.365	93.109	100.889

**Table 3:** Experiment 3 (the weight is 106.8 kg).

**Loading Force/N**	−0.137	16.088	53.096	99.811	148.805	199.85
**Output Force 1/N**	0	8.675	26.58	49.16	73.094	97.952
**Output Force 2/N**	0	6.713	25.16	48.249	72.906	98.442

**Table 4:** Experiment 4 (the weight is 176.1 kg).

**Loading Force/N**	0.003	18.777	58.793	103.457	151.312	198.164
**Output Force 1/N**	−0.013	12.121	31.133	52.544	75.802	98.321
**Output Force 2/N**	0.015	4.517	24.469	46.68	70.835	94.426

**Table 5:** Coupling degree of the branches.

i	1	2	3	4
$f_{L}$ (KN)	2.212	1.312	3.485	2.998
$f_{M}$ (KN)	2.186	1.275	3.455	2.996
ε (%)	1.18%	2.85%	0.86%	0.1%

**Table 6:** Mesh data of the sensor.

Unit Type	Unit Number	Number of Nodes
Solid187	90,752	147,965

**Table 7:** Axial deformations of the four branches.

Loading Force/N	*D*1/mm	*D*2/mm	*D*3/mm	*D*4/mm
300	6.45E−4	3.14E−4	3.11E−4	1.18E−6
600	1.29E−3	6.28E−4	6.23E−4	2.37E−6
900	1.94E−3	9.42E−4	9.34E−4	3.56E−6
1200	2.58E−3	1.26E−3	1.25E−3	4.75E−6
1500	3.23E−3	1.57E−3	1.56E−3	5.93E−6
1800	3.87E−3	1.88E−3	1.87E−3	7.12E−6
2100	4.52E−3	2.20E−3	2.18E−3	8.31E−6
2400	5.16E−3	2.51E−3	2.49E−3	9.50E−6
2700	5.81E−3	2.83E−3	2.80E−3	1.07E−5
3000	6.45E−3	3.14E−3	3.11E−3	1.19E−5
3300	7.10E−3	3.45E−3	3.43E−3	1.31E−5

**Table 8:** Axial deformations of the four branches.

Loading Force/N	*D*1/mm	*D*2/mm	*D*3/mm	*D*4/mm
300	−1.96E−5	−1.98E−5	−1.94E−5	−4.06E−4
600	−3.90E−5	−3.95E−5	−3.87E−5	−8.12E−4
900	−5.85E−5	−5.93E−5	−5.80E−5	−1.22E−3
1200	−7.83E−5	−7.90E−5	−7.74E−5	−1.62E−3
1500	−9.73E−5	−9.87E−5	−9.67E−5	−2.03E−3
1800	−1.13E−4	−1.18E−4	−1.16E−4	−2.44E−3
2100	−1.36E−4	−1.38E−4	−1.35E−4	−2.84E−3
2400	−1.50E−4	−1.58E−4	−1.55E−4	−3.25E−3
2700	−1.70E−4	−1.78E−4	−1.74E−4	−3.66E−3
3000	−1.95E−4	−1.97E−4	−1.93E−4	−4.06E−3
3300	−2.13E−4	−2.17E−4	−2.13E−4	−4.47E−3

## Abbreviations

12-SS	A kind of mechanism configuration. S-spherical pair
FEM	Finite Element Method
BP	Back Propagation
RBF	Radical Basis Function
